# A Treatise on the Mineral Waters of Harrogate and Its Vicinity

**Published:** 1831

**Authors:** 


A TREATISE ON THE MINERAL WATERS OF HARROGATE AND ITS VICINITY. By *Adam Hunter*, M.D. &c. Small octavo, pp. 138. 1830.

THE experience of ten years in the vicinity of Harrogate, during which great numbers of people have taken the waters under Dr. Hunter's directions, renders him every way calculated for the present undertaking, the object of which is, to afford a complete analysis of all the mineral springs hitherto discovered there, which are either medicinally important, or remarkable for their situation or contents, together with succinct directions for their use. Although we do not question the medicinal powers of the Harrogate waters, we doubt the soundness of the conclusion, that—"no stronger proof can be afforded of the great efficacy of the waters, than the resort of such a number of visitors." This is precisely the proof which St. John Long and his disciples (for that is a more proper designation than patients) offer for the truth of his miraculous cures—the efficacy of his wonder-working liniment. What happens to a doctor may happen to a watering-place. Many have fancied themselves cured by the waters, when it was to change of air and scene, together with exercise, that they owed their recovery. But still they would praise the spot where health was obtained, and recommend it in all diseases among their neighbours.

We must pass over the history of these medicinal springs, which may amuse a dull hour while loitering at Harrogate, but which do not require notice here.

These springs are now become so numerous, that it is necessary to divide them into four classes.

"Class I. Springs impregnated with sulphuretted hydrogen gas and saline matter.
II. Saline chalybeate springs.
III. Pure chalybeate springs.
IV. Springs containing earthy salts, with little iron and no sulphuretted hydrogen gas." 23.

1. *Old Sulphur Well.* This, of course, is in the first class. It is perfectly clear—temperature about 49°—smell highly sulphureous—taste sulphuretted and strongly saline (a mixture of flavours to which the palate is soon reconciled)—deposites a white sediment on standing, and loses its smell. By the various tests employed, it was ascertained that this water contains chlorine, in combination with the bases of lime, magnesia, and soda—no sulphuric acid, no iron.

2. *Oddy's Saline Chalybeate, or Cheltenham Spring.* This spring is considered so important in its nature and effects as to be placed in a class by itself. It was discovered in the Autumn of 1818. When taken from the spring, it is transparent and has a sparkling appearance—taste distinctly chalybeate, and considerably saline. Ten years ago, Dr. Hunter published some account of this water, and suggested the diseases in which it would be beneficial. The subsequent experience of the author has confirmed the opinions then broached. Dr. H. combats certain positions laid down by Dr. Scudamore; but these controversies we shall pass over. The imperial gallon of this water contains—

| | |
|---|---|
| Of Muriate of Soda | 360·48 |
| Muriate of Lime | 26·4 |
| Muriate of Magnesia | 11·88 |
| Sulphate of Lime | 2·23 |
| Carbonate of Lime | 8·04 |
| Carbonate of Magnesia | ·96 |
| Oxide of Iron | 2·88 |
| Residue, consisting chiefly of Silex | ·48 |
| | 413·35 |

The following passage we shall insert.

"It may be taken with proper management either as a tonic, an alterative, or aperient, and therefore becomes more especially useful in a wide range of complaints connected with biliary derangement and atony of the stomach. There are many who, after having taken the sulphur water at Harrogate, are recommended to proceed to Cheltenham, a journey unpleasant to some and inconvenient as well as expensive to others. This step, so far at least as concerns the relative properties of the waters, is evidently unnecessary, and may be entirely superseded by a similar course of this water upon the spot."

3. *Chalybeate Springs.* The old or sweet spa, as it is called, has for some years been the principal chalybeate used at Harrogate. Although there are not more than two grains of iron in a gallon of this water, it is a perfect wonder-worker in the cure of diseases. The following testimony, carefully re-printed by Dr. Hunter, will put *Singeing* Long's liniment to the blush.

"As to the virtues of this spring, there is scarce any disease incident to mankind wherein its inward or outward use may not be of service. I have been an eye witness of its effects nearly forty years and I have not neglected drinking it myself any one season all that time; and though I am now in my 66th year, yet I am strong and vigorous, free from the complaints of old age. But because a general and just commendation of this spring will not be satisfactory, without condescending to enumerate the diseases wherein it's proper. It's good, therefore, to restore a lost Appetite and Digestion, to mitigate the Scurvy, correct all *acid Humours* in the Lympha, Blood, *nervous* and pancreatic Juices. It cleanses the Kidnies and Ureters of Slime, Sand, Gravel and *great Stones*, and is very assistant in curing Ulcers in those Parts. It removes the Hyppo's Melancholy, opens Obstructions of the Lungs, Liver, Spleen, Mesentery and Glands. It purifies the Blood, and renders the Spirits in the Body more *cheerful and lively*. Several short-winded, Asthmatic, weak and lame People, have had their Lungs and Limbs restored to their former strength and usefulness. It relieves inveterate Head-Aches, especially if at the same time you use the Cold Bath. It is also very serviceable in the Gout, by restoring the use of lame Hands, Knees, Legs and Feet. It *revives the Memory*, clears the Brain from viscous Humours, and helps the Eyes by drying up Rheums. It relieves sharpness of Urine, Strangury and Dysury, if there is no large Stone or other stoppage in the urinary passages. It corrects Acidity in any part of the Body; as in the Heartburn, Belchings, Sourness at the Stomach, Gripes, Cholic, and Borborigmos. It opens the Breast and Lungs, *cuts tough Flegm*, promotes Expectoration, and has often been successful in the Cure of Blood-spitting, Hectic Fever, too great Heat and Dryness of the Skin and Body."* 66.

"This (says Dr. Hunter) is a tolerably fair specimen of *a few* of the miseries of our ancestors which were cured or alleviated by the use of this water."

---

* Dr. Neal, of Leeds.

Speaking of the TEWIT WELL, Dr. Hunter informs us that it was the first of the mineral waters discovered at Harrogate, and was frequented, 260 years ago, by "innumerable herds of people." Volumes have been written upon the surprising cures performed by it—cures that are stated to have been "the most remarkable filed up in the *authentic records* of physic." Yet, strange to say, although its waters run "pure, translucid, and unimpaired, it is now almost entirely neglected." We are a little surprized to find so sensible a man as Dr. Hunter fill up so much of his book with idle stories, published one or two centuries ago, respecting the miracles performed by the Harrogate Waters.

4. The *Saline Springs* occupy only two or three pages of our author's work. Dr. Garnett states that the water of the CRESCENT OLD WELL contains 13 cubic inches of sulphuretted hydrogen gas, and two grains of carbonate of iron to the gallon. It appears, however, that the water contains no carbonate of iron.

The sulphur and saline waters of Harrogate, especially when combined with the external application of the former, are the most important of the springs. We can only afford space for one more extract.

"The Sulphur and Saline Waters are taken with greatest advantage at the well, in the morning before breakfast, using gentle exercise between the intervals of drinking. A glass containing half a pint should be taken, and repeated once or twice at an interval of fifteen minutes or half an hour. In a great majority of cases this will be found sufficient. But when the bowels are more than usually constipated from previous disease, or any other cause, a larger quantity is required, and two or three pints may be taken not only with safety but advantage. These waters were used by all ranks in former times, and by the lower orders to this day, in larger quantities than is here recommended. The cures have sometimes been very surprising, but the bad effects arising from such immoderate doses have likewise been sufficiently serious.

Some practitioners are accustomed to recommend a mercurial, or other aperient pill, to be taken during the whole course of the sulphur water; however beneficial this practice may be at the commencement, I consider it in most instances unnecessary after the action of the water is established, and the system becomes reconciled to its effects. By those using the pure chalybeate water, aperient medicine is frequently required during the whole course. As a general rule, the aperient taken should coincide as nearly as possible with the intention of the water, and the removal of the disease.

There are many who suffer considerable uneasiness from the quantity of cold water taken into the stomach. The most delicate invalids, and those, on the other hand, to whom pure water in any form is a rare beverage, are the greatest sufferers from this cause. To obviate these distressing sensations, the doses of the water should either be small, and repeated at longer intervals, or a portion of it made hot, should be added to each draught.

This is found to be more frequently necessary for those using the saline chalybeate. As these waters lose part of their medicinal powers by being heated, it is better to add a portion of hot water to the cold, at the moment when it is taken, than that the whole should be exposed to the action of fire. When this is found insufficient a teaspoonful or two of some light spirit or aromatic tincture may be added to the water." 86.

Dr. Hunter has appended many judicious remarks on the method of employing the Harrogate waters, and the rules of Hygiene to which invalids should submit, if they expect to derive full benefit from the watering-place in question. These directions and remarks are only calculated for non-professional invalids, and need not be noticed in a medical journal.

